# Influence of Different Previous Frozen Holding Periods on the Canned Fish Quality

**DOI:** 10.3390/foods12224117

**Published:** 2023-11-13

**Authors:** Elena Villamarín, Beatriz Martínez, Marcos Trigo, Santiago P. Aubourg

**Affiliations:** 1 Department of Food Technology, Marine Research Institute (CSIC), c/Eduardo Cabello, 6, 36208 Vigo, Spain; elenavlop22@gmail.com (E.V.); mtrigo@iim.csic.es (M.T.); 2 Department of Food Technologies, CIFP Coroso, Avda. da Coruña, 174, 15960 Ribeira, Spain; bmartinezr@edu.xunta.gal

**Keywords:** horse mackerel, canning, cold storage, lipid oxidation, lipid hydrolysis, fatty acid profile, colour changes, trimethylamine, degradation

## Abstract

The combined effects of thermal processing (i.e., sterilisation treatment) and the prior frozen storage time (3 or 6 months at −18 °C) on the quality loss in canned Atlantic horse mackerel (*Trachurus trachurus*) were determined. Thus, the sterilisation step led to a remarkable (*p* < 0.05) formation in the canned fish muscle of fluorescent compounds, free fatty acids (FFAs), and trimethylamine and an increase in the L* and b* colour parameters; meanwhile, a decrease (*p* < 0.05) in the total ω3 FA/total ω6 FA ratio and the a* colour value were detected. The prior frozen storage period led to an increased (*p* < 0.05) lipid oxidation (peroxide, thiobarbituric acid reactive substance, and fluorescent compound formation) and hydrolysis (FFA formation) development and to increased L* and b* colour values in the corresponding canned samples; additionally, a lower (*p* < 0.05) polyene index and phospholipid content were observed in canned fish previously subjected to frozen storage. In most indices, physico-chemical changes related to quality loss were found to be higher if the previous storage period was increased. According to the marked effects of the sterilisation step and the prior frozen storage period, the optimisation of such processing conditions is recommended to maximise the quality of canned horse mackerel.

## 1. Introduction

Canning procedures can preserve marine species via the combination of sealing in a hermetic container and heating to destroy spoilage and pathogenic microorganisms and inactivate enzymes [1,2]. Provided that duration and temperature during the heating and cooling cycle of the thermal process are properly carried out, canned seafood can be preserved for a long time. However, long-term high temperatures may cause losses of quality in the nutritional value (destruction of vitamins and protein components) and loss in the sensory quality, such as the soft texture, the separation of jelly and fat, discolouration, and undesirable taste [3,4]. Therefore, the search for optimised canning conditions [5] and the employment of advanced preserving technologies [6] was found to be necessary.

According to the short shelf-life time of refrigerated marine species, excess raw material is frozen prior to canning. Freezing followed by frozen storage is considered a valuable alternative for relatively long preservation and has been used increasingly [7,8]. In most cases, marine species can be preserved at times of glut and stored under frozen conditions until supplied to canneries. However, measurements of sensory, chemical, and physical changes have shown that the deterioration of fish quality continues to some extent during the frozen storage since undesirable changes associated with lipids and proteins are produced [9,10]. As a result, most concerns with canned fish have been shown to be related to the quality of the raw material employed. Thus, previous studies have shown a relevant effect of time [11,12] and temperature [13] of prior cold storage on the quality of the resulting canned product. However, previous research regarding the combined effect of prior frozen storage and subsequent canning processes can be considered scarce.

Atlantic horse mackerel (*Trachurus trachurus*), also known as European horse mackerel or common scad, is a pelagic fish species found in the eastern Atlantic Ocean of Europa and Africa and in the South-Eastern Indian Ocean [14,15]. Previous research regarding quality changes in this species during processing has been focused on fresh and frozen products [16,17]. Thus, different preserving strategies have been developed for quality enhancement and for the shelf-life time increase under storage conditions, such as the addition of natural preserving extracts [18,19], biopreservation [20], irradiation [21], and high-pressure [22]. Although recent research has addressed the quality enhancement of brine-packed horse mackerel via the addition of an antioxidant extract to the packing medium [23], it can be concluded that previous research on canned horse mackerel is very scarce.

The present study focused on the quality of canned horse mackerel (*T. trachurus*). Its basic objective was to analyse the influence of different previous frozen holding periods on the physico-chemical changes related to quality loss of the canned product. For this, complementary quality indices such as lipid oxidation and hydrolysis development, changes in fatty acid (FA) composition and colour parameters, and trimethylamine (TMA) formation were determined in raw and canned samples.

## 2. Materials and Methods

### 2.1. Raw Fish and Fish Processing

A total of 32 horse mackerel fish (weight range: 165–185 g; length range: 27–31 cm) were obtained in winter 2022 at Vigo Harbour (North-Western Spain) and transported on ice to the laboratory. Upon arrival at the laboratory, eight individuals were selected, divided into four batches (two individuals per batch) and considered raw fish. The fish individuals were beheaded, eviscerated, and filleted, and the dark muscle was discarded. Within each batch, the white muscle was pooled together, minced, and analysed independently (*n* = 4).

On the same day, a second group of eight fish individuals was taken, divided into four batches (two individuals per batch), beheaded, eviscerated, filleted, and subjected directly to the canning process according to the conditions expressed later on. Such canned samples were labelled as not previously frozen (0-month samples).

The remaining sixteen fish were placed in individual flexible polyethylene bags, kept at −40 °C for 48 h, and then stored at −18 °C for 3 or 6 months. At each storage period, eight individuals were thawed overnight (4 °C), separated into four batches (two individuals per batch), beheaded, eviscerated, filleted and subjected to the canning procedure according to conditions expressed later on. The resulting samples were labelled as canned samples with prior 3- or 6-month frozen storage (3-month and 6-month samples), respectively.

At each canning time, 45 g portions of horse mackerel fillets (from one fish) were introduced in small flat rectangular cans (105 × 60 × 25 mm; 150 mL). Then, the cans were filled with 90 mL of distilled water, vacuum-sealed (SOMME 222, Ezquerra, San Adrián, Navarra, Spain), and sterilised (115 °C, 45 min; *F*_o_ = 7 min) (CIFP Coroso, Ribeira, A Coruña, Spain) in a steam retort (Presoclave II 75L, JP Selecta, Barcelona, Spain). When the heating time was accomplished, the steam was cut off, the remaining steam was flushed away by the use of air, and water at reduced pressure was employed for cooling the cans.

The cans were opened after 3 months of storage at room temperature (20 °C). Then, the liquid part was carefully drained off gravimetrically and filtered through a filter paper and the dark muscle was discarded. The white muscle of horse mackerel was wrapped in filter paper and used for analysis. For each sample, the fish muscle corresponding to two cans was pooled together and considered for carrying out the different physico-chemical analyses. Four different batches of canned samples were analysed independently (*n* = 4). Additionally, each physico-chemical analysis was carried out in triplicate in each single sample.

In agreement with common practice employed in canneries, a 3-month storage was employed. Commonly, manufacturers indicate that a minimum of a 2–3-month storage is required to optimise the acceptability of commercial canned fish [24].

All chemical reagents and solvents employed were of reagent grade (Merck, Darmstadt, Germany).

### 2.2. Lipid Oxidation Assessment

The extraction of the lipid fraction was carried out on the fish’s white muscle by employing the Bligh and Dyer [25] procedure. This method is based on a single-phase solubilisation of the lipids with a chloroform–methanol (1:1) mixture. The results were expressed as g lipid·kg^−1^ muscle. 

The peroxide value (PV) was assessed via spectrophotometric analysis (Beckman Coulter, DU 640; London, UK) on the lipid extract. For this, peroxide reduction with ferric thiocyanate was employed, following the Chapman and McKay [26] method. The results were calculated as meq active oxygen·kg^−1^ lipids.

The thiobarbituric acid index (TBA-i) was assessed in agreement with the Vyncke [27] procedure. For this, the reaction between thiobarbituric acid reactive substances (TBARS) present in a trichloroacetic acid extract of the fish muscle and thiobarbituric acid was carried out. Determination of TBARS content was accomplished spectrophotometrically at 532 nm and calculated from a standard curve prepared with 1,1,3,3-tetraethoxy-propane (TEP). The results were calculated as mg malondialdehyde·kg^−1^ muscle.

### 2.3. Assessment of Fluorescent Compounds

The formation of interaction compounds between oxidised lipids and nucleophilic compounds (i.e., protein-like molecules) was assessed via fluorescence spectroscopy (Fluorimeter LS 45; Perkin Elmer España; Tres Cantos, Madrid, Spain). In agreement with previous research [11,28], fluorescence was measured at excitation/emission of 393/463 and 327/415 nm in the lipid extract of the fish muscle. The relative fluorescence (*RF*) was calculated as follows: *RF* = *F*/*Fst*, where *F* is the fluorescence measured at each excitation/emission wavelength pair, and *Fst* is the fluorescence intensity of a quinine sulphate solution (1 µg·mL^−1^ in 0.05 M H_2_SO_4_) at the corresponding wavelength pair. The fluorescence ratio (*FR*) was calculated as the ratio between the two *RF* values: *FR* = *RF*_393/463 nm_/*RF*_327/415 nm_.

### 2.4. Determination of Free Fatty Acid (FFA) and Phospholipid (PL) Content

FFA content was assessed in the lipid extract of horse mackerel muscle according to the Lowry and Tinsley [29] procedure. This method implies a complex formation of FFA with cupric acetate–pyridine, followed by spectrophotometric (715 nm) determination. For quantitative purposes, oleic acid was employed as standard. The results were calculated as g FFAs·kg^−1^ lipids.

PL content was determined by measuring the organic phosphorus in the total lipid extracts in agreement with the Raheja et al. [30] procedure. This method implies a complex formation of organic phosphorus with ammonium molybdate. For quantitative purposes, 1,2-dipalmitoyl-rac-glycero-3-phosphocholine was employed as standard. The results were expressed as g PLs·kg^−1^ muscle and as g PL·kg^−1^ total lipids.

### 2.5. Analysis of FA Composition

Fatty acid methyl esters (FAMEs) from lipid extracts were obtained by employing acetyl chloride in methanol. Then, analysis of FAMEs was carried out via gas–liquid chromatography (PerkinElmer 8700 chromatograph; Madrid, Spain) in agreement with an established procedure [31]. The resulting FAME peaks were identified by comparing their retention times with those of standard mixtures (Qualmix Fish, Larodan, Malmo, Sweden; FAME mix, Supelco, Inc., Bellefonte, PA, USA). For quantitative purposes, peak areas were automatically integrated, and C19:0 FA was used as an internal standard. The content of each FA was expressed as g·100 g^−1^ total FAs.

Results regarding total ω3 FAs, ω3/ω6 ratio and polyene index (PI) (C20:5ω3 + C22:6ω3/C16:0 ratio) were calculated taking into account the values of the corresponding individual FAs.

### 2.6. Other Physico-Chemical Determinations

Colour parameters (L*, a* and b*) were determined on the surface of the raw and canned fish muscle. For this, instrumental colour analysis (CIE 1976 Lab), performed with a tristimulus Hunter Labscan 2.0/45 colourimeter, was carried out. For each sample analysis, colour scores were averaged over four determinations, which were taken by rotating the measuring head 90° between triplicate measurements per position.

TMA content was determined using the picrate colourimetric (Beckman Coulter, DU 640; London, UK) method, as previously described by Tozawa et al. [32]. This method involves the preparation of a 5% trichloroacetic acid extract of fish muscle (10 g/25 mL). The results were expressed as mg TMA-N·kg^−1^ muscle.

### 2.7. Statistical Analysis

As expressed above, four replicates (*n* = 4) were considered in this study. Data obtained were evaluated by analysis of variance (ANOVA) to explore differences resulting from the effect of the sterilisation step and the prior frozen storage time. For carrying out mean values comparison, the least-squares difference (LSD) procedure was developed. A confidence interval at the 95% level (*p* < 0.05) was considered in order to establish significant differences among batches in all instances. For this, the PASW Statistics 18 software for Windows (SPSS Inc., Chicago, IL, USA) was employed. Correlation values among the different quality indices were determined by the Pearson test.

## 3. Results and Discussion

### 3.1. Lipid Oxidation Development

The lipid content of raw horse mackerel was 14.17 ± 2.41 g lipid·kg^−1^ muscle. With the aim of obtaining an accurate analysis of the lipid oxidation development in the current fish processing study, different and complementary methods, i.e., peroxide, TBARS and fluorescent compound determinations, were carried out.

A comparison of peroxide levels in raw fish and canned fish not previously frozen showed no effect (*p* > 0.05) of the sterilisation process on this kind of lipid oxidation compound (Table 1). However, a marked increase (*p* < 0.05) was recorded in canned fish frozen stored for 3 or 6 months, proved by including a prior frozen storage time. Notably, no differences (*p* > 0.05) were detected by comparison of canned samples corresponding to 3 and 6 months of prior frozen storage.

A similar result was observed for the evolution of the TBARS content (Table 1). Thus, although an increased average TBARS value was proved after the heating step, differences were not significant (*p* > 0.05). Additionally, canned fish corresponding to prior frozen storage (i.e., 3 and 6 months) showed higher (*p* < 0.05) levels of TBARS than the raw fish and the canned fish that were not subjected to frozen storage. As for the peroxide determination, no effect (*p* > 0.05) on the TBA-i was recorded by increasing the prior frozen storage from 3 to 6 months.

Contrary to peroxide and TBARS values, the FR showed a marked increase (*p* < 0.05) as a result of the sterilisation step (Table 1). Additionally, FR values increased (*p* < 0.05) if a prior frozen storage period (i.e., 3 or 6 months at −18 °C) was included. As for peroxide and TBARS determinations, the highest average values were detected in canned fish previously subjected to the longest storage period; however, no significant differences (*p* > 0.05) with samples corresponding to 3-month storage were detected.

The oxidation of the lipid fraction in seafood is considered a complex deteriorative mechanism. Thus, it involves the formation of a great diversity of molecules, most of them unstable and therefore susceptible to breakdown, and leads to the formation of lower-weight compounds, which in turn can react with nucleophilic-type molecules (peptides, free amino acids, proteins, etc.) present in the fish muscle [3,33]. In the present study, the heat treatment (i.e., sterilisation) favoured the formation of interaction compounds with fluorescent properties, according to the development of two damage mechanisms (i.e., non-enzymatic lipid oxidation and non-enzymatic browning) [16]. Additionally, the formation of lipid oxidation compounds should also be produced during the frozen storage period as a result of the endogenous enzyme (i.e., lipoxygenases, peroxidases, etc.) activity and autolysis [9,10]; this oxidant effect should increase with the storage duration and temperature. Values obtained in the present research were in all cases included in the 1.36–5.91 (PV) and 0.04–0.32 (TBA-i) ranges; therefore, a relevant value of primary and secondary lipid oxidation compounds was not obtained and could be explained on the basis of being relatively unstable molecules [33,34].

The assessment of the fluorescent compounds produced as a result of the interaction between lipid oxidation compounds and nucleophilic-type molecules present in the fish muscle has shown to be a highly valuable tool in the present study to describe changes occurring as a result of the sterilisation step and the previously frozen storage. Fluorescence spectroscopy has already proved to be a valuable tool for the discrimination of fresh and frozen fish [35], for fish authenticity [36], and for the assessment of lipid damage during processing [11,28].

A low peroxide content was observed in different canned fish species such as olive oil-packed bluefin tuna (*Thunnus thynnus*) and tomato sauce-packed sardine (*Sardina pilchardus*) [37], sunflower oil- and olive oil-packed eel (*Anguilla anguilla*) [38], and brine-packed [12] and water-packed [39] Atlantic Chub mackerel (*Scomber colias*). In such studies, the low primary oxidation compound content was explained on the basis that thermal treatment would partially destroy such kinds of molecules and lead to the formation of low molecular weight molecules (i.e., carbonyl compounds). Additionally, the interaction of lipid oxidation compounds with nucleophilic molecules present in the muscle may lead to the formation of fluorescent compounds [11,40]. Contrary to the present results, an increase in the peroxide presence in canned fish as a result of the sterilisation process was observed in brine-canned mackerel (*S. colias*) [41].

Previous investigations concerned a great number of studies focused on the effect of canning on the TBARS content of canned fish. In most cases, an increase in the TBARS content has been described in canned fish when an oil-packing medium was employed. On the contrary, aqueous-packing media have led either to non-different values or to a decrease in the TBARS level in canned fish. Not detecting a TBARS content increase has been explained on the basis of TBARS leaching into the aqueous packing medium. Thus, an increased formation was observed in canned sardine (*S. pilchardus*) by employing brine- and olive oil-packing [11]; in sunflower-, groundnut-, and coconut-packed yellowfin tuna (*Thunnus albacares*) [42]; in sunflower oil-, olive oil-, soybean oil-, and brine-packed silver carp (*Hypophthalmichthys molitrix*) [43]; and in sunflower oil- and olive oil-packed eel (*A. anguilla*) [38]. On the contrary, a marked decrease in the TBARS value was described for tomato sauce-packed sardine (*S. pilchardus*) [37] and brine-packed Atlantic Chub mackerel (*S. colias*) [12]. In agreement with the present results, no effect on the TBARS content was observed in water-packed mackerel (*S. colias*) [44].

Previous seafood research has shown a marked increase in the FR as a result of the sterilisation process. In fact, this analytical measurement was proposed as a lipid quality index for canned fish [11]. In agreement with the current results, an increased fluorescent compound formation was detected in varied canned fish products such as sunflower oil-, olive oil-, soybean oil-, and brine-packed silver carp (*H. molitrix*) [43]; brine-packed Atlantic Chub mackerel (*S. colias*) [12,41]; water-packed Atlantic mackerel (*S. scombrus*) [45]; and water-packed Atlantic Chub mackerel (*S. colias*) [39].

Regarding the effect of the prior frozen storage on the lipid oxidation development in canned seafood, a general increase in the FR value was reported in canned fish with an increased prior frozen period. These results include olive oil-packed sardine (*S. pilchardus*) (prior to 0–12-month period at −18 °C) [11], brine-packed Atlantic Chub mackerel (*S. colias*) (0–15-month period at −18 °C) [12], and brine-canned horse mackerel (*T. trachurus*) (0–6-month period at −18 °C) [23].

### 3.2. Lipid Hydrolysis Development

**Figure 1:**
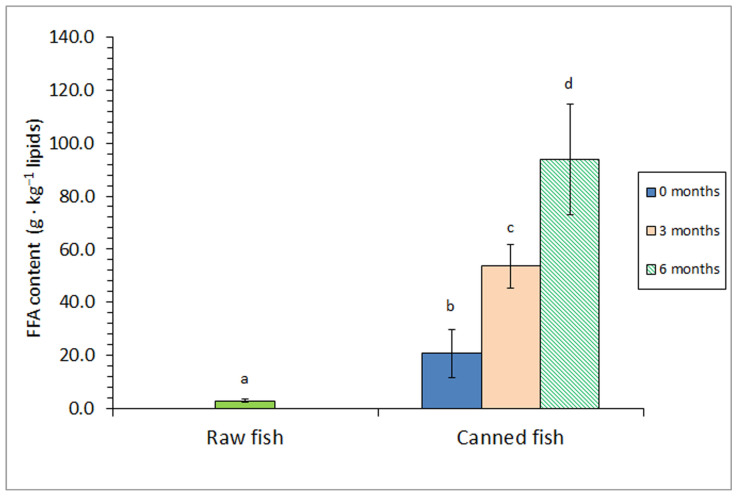
Free fatty acid (FFA) content of raw and canned horse mackerel previously subjected to different frozen storage times (0, 3, and 6 months). Mean values of four (*n* = 4) replicates. Standard deviations are denoted by bars. Different letters (a–d) denote significant differences (*p* < 0.05).

A comparison of the FFA content in raw fish and canned fish without a prior frozen period showed a strong formation of this kind of molecule resulting from the heating process (Figure 1). Furthermore, a subsequent increase (*p* < 0.05) in the FFA presence was proved by including a prior frozen storage; this increase was more important (*p* < 0.05) if the storage period was increased.

**Figure 2:**
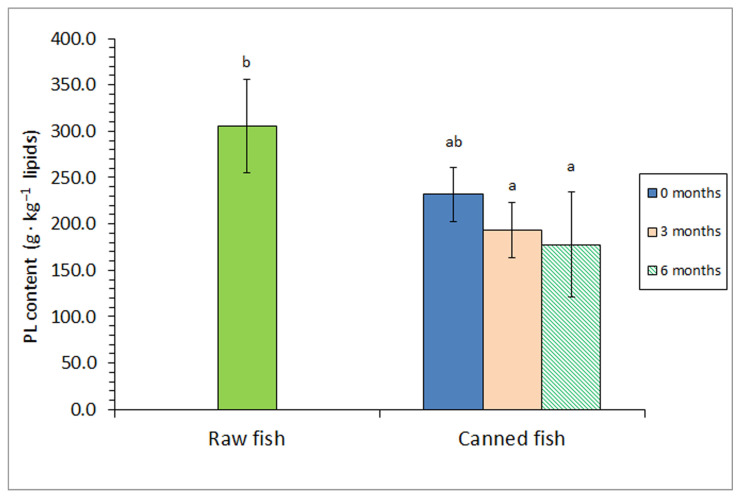
Phospholipid (PL) content of raw and canned horse mackerel previously subjected to different frozen storage times (0, 3, and 6 months). Mean values of four (*n* = 4) replicates. Standard deviations are denoted by bars. Different letters (a, b) denote significant differences (*p* < 0.05).

Contrary to FFA results, an average decrease could be observed for the PL content as a result of the sterilisation step (Figure 2); however, this decrease was not found significant (*p* > 0.05). If a prior frozen storage period was included (i.e., 3 or 6 months at −18 °C), canned samples depicted lower (*p* < 0.05) PL values than those reported for the raw fish. The evolution of the PL content in the lipid fraction showed a high inverse correlation with the FFA presence (*r* = −0.924, logarithmic fitting; *p* < 0.05).

FFA is considered to be the result of the hydrolysis of high molecular weight lipid compounds such as triacylglycerols (TAGs) and PLs. In the present study, FFA content can be considered the result of different factors. First, the sterilisation process can lead to hydrolysis of lipid classes such as TAGs and PLs as a result of thermal breakdown (i.e., non-enzymatic lipid hydrolysis) [11,44]. On the other side, FFA formation is reported to be produced during the frozen storage by the action of endogenous enzymes (phospholipases and lipases in general) present in the fish muscle; this effect showed to increase with the storage duration and temperature [9,10]. Remarkably, FFA accumulation was accepted as not having nutritional significance. Nevertheless, it was recognised as being involved in several deteriorative mechanisms during seafood processing, leading to off-taste and off-odour development, muscle texture changes, and the acceleration of the formation of lipid oxidation compounds [10,33].

Regarding PL compounds, this group of lipids has attracted great attention for showing a high bioavailability and preserving effect on several diseases and for delivery systems [46,47]. Based on industrial requirements (food production and pharmaceutical industries), important efforts are being addressed for the retention of PL constituents from seafood and their corresponding by-products as presenting high PUFA levels [48,49].

Previous research reported on the effect of canning on the FFA content of canned fish muscle. Thus, and in agreement with the present results, sunflower oil- or brine-packed sprat (*Clupeonella cultriventris*) [50], water-canned Chub mackerel (*S. colias*) [39], and brine-canned Chub mackerel (*S. colias*) [41] revealed a marked FFA formation when compared to the starting raw material. Notably, an important effect of the filling medium on the FFA value has been reported in previous research. Thus, Naseri et al. [43] showed an FFA content increase in canned silver carp (*Hypophthalmichthys molitrix*), including olive oil, sunflower oil, soybean oil or brine as packing media. Additionally, the employment of sunflower oil increased the preservative effect of processed yellowfin tuna (*T. albacares*) against FFA formation when compared to groundnut and coconut oils as packing media [42].

Regarding the effect of the sterilisation process on the PL value in canned seafood, previous studies can be considered scarce. In agreement with the current results, a marked loss of PL compounds could be observed in the brine-canned Chub mackerel (*S. colias)* muscle [44]. This loss could be explained on the basis of different mechanisms. First, since they show a high PUFA level [48,49], PLs could be damaged easily by the heating treatment. Additionally, PL classes may be partially hydrolysed during the prior frozen storage period via endogenous enzyme activity [9,10]. Finally, as a group, including relatively polar lipid compounds, PLs could be lost partly by leaching from the muscle into the packing medium [44].

Previous studies regarding the influence of a prior frozen storage period on the FFA and PL contents in canned seafood can be considered scarce. According to the current results, Prego et al. [12] proved an increase in the FFA content in brine-canned Atlantic Chub mackerel (*S. colias*) when an increased prior frozen period (0–15 months at −18 °C). Additionally, Méndez et al. [23] observed an FFA value increase and a PL presence decrease in brine-canned horse mackerel (*T. trachurus*) if a frozen period (0–6-month period) was previously employed.

### 3.3. Fatty Acid Analysis

The FA analysis of the lipid fraction corresponding to the raw fish indicated the following profile (g·100 g^−1^ total FAs): 4.24 ± 0.76 (C14:0), 0.54 ± 0.03 (C15:0), 22.15 ± 0.10 (C16:0), 4.42 ± 0.29 (C16:1ω7), 1.22 ± 0.09 (C17:0), 7.52 ± 0.16 (C18:0), 15.88 ± 1.68 (C18:1ω9), 3.07 ± 0.17 (C18:1ω7), 1.46 ± 0.06 (C18:2ω6), 1.70 ± 0.30 (C20:1ω9), 0.32 ± 0.03 (C20:2ω6), 1.45 ± 0.11 (C20:4ω6), 0.25 ± 0.03 (C22:1ω9), 7.67 ± 0.04 (C20:5ω3), 0.43 ± 0.04 (C22:4ω6), 0.75 ± 0.08 (C24:1ω9), 2.89 ± 0.11 (C22:5ω3), and 23.78 ± 2.82 (C22:6ω3).

In agreement with the profile obtained, DHA, C16:0, C18:1ω9, EPA, and C18:0 showed to be the major FAs in the present fish species. In recent decades, great interest has been attributed to seafood according to its beneficial health effects. In agreement with recent reports [51,52], the total value of ω3 unsaturated FAs is considered a highly valuable index. According to clinical and epidemiological research, EPA consumption has been associated with an inhibitory effect on the development of inflammatory, coronary, and circulatory diseases [53]. Regarding DHA, it has been associated with foetal development, the prevention of neurodegenerative diseases, and the appropriate functioning of the nervous system and visual organs in the foetus [54]. In this context, great attention was directed to the ω3/ω6 ratio of foods included in the human diet [55,56]. With the aim of preventing relevant health concerns, values included in the 0.25–1.00 range have been recommended for the ω3/ω6 ratio [57]. According to such nutritional and healthy properties, discussion on FA results obtained in the present study will now be focused on the content of ω3 FAs and PUFAs in the lipid fraction. The evolution of their content during the thermal treatment and the previous frozen storage period would be closely related to the above-mentioned development of lipid oxidation and hydrolysis in the fish muscle.

Small differences (*p* > 0.05) could be observed for the EPA content among the different samples considered in the current study (Table 2). Thus, an increased average value was depicted resulting from the sterilisation step; however, differences between raw fish and canned samples without a prior storage period were not found significant (*p* > 0.05). Canned samples corresponding to a 3-month storage period showed the highest average value, which was found to be significantly higher (*p* < 0.05) than the one reported for the raw fish; however, comparison to other canned samples (without prior storage period and with a 6-month period) did not show significant differences (*p* > 0.05).

Regarding the DHA value, the raw fish showed higher average values than any of the canned samples considered in the present study (Table 2). Remarkably, differences were found significant (*p* < 0.05) when compared to canned fish that were kept under frozen conditions, but not (*p* > 0.05) when compared to canned samples not previously stored. Additionally, the lowest average DHA content was found in samples corresponding to the longer prior storage time. 

In a recent study, Prego et al. [31] analysed the effect of packing media (water, brine, sunflower oil, refined olive oil, and virgin olive oil) on canned Atlantic mackerel (*S. scombrus*). As a result, EPA content showed to increase when employing water and brine as packing media; DHA presence increased in canned mackerel packed in refined olive oil and virgin olive oil; the total ω3 FA value increased in canned fish when water, sunflower oil, refined olive oil, and virgin olive oil were used as filling media; and the PUFA/STFA ratio value increased under all packing conditions. On the contrary, no influence on the ω3/ω6 ratio value was obtained under any of the packing conditions tested.

Previous studies regarding the influence of prior frozen holding on the FA composition of canned marine species are scarce. No remarkable influence was observed in the PI of olive oil-canned sardine (*S. pilchardus*) muscle with increased prior storage time (12 months at −18 °C) [11]. On the contrary, a PI decrease in brine-canned Chub mackerel (*S. colias*) was observed by increasing the prior frozen period (0–15-month period) [12]. Additionally, a similar decreasing tendency for the PI was reported by Méndez et al. [23] in brine-canned horse mackerel (*T. trachurus*) previously stored for a 0–6-month period under frozen conditions. Recently, Prego et al. [31] studied the influence of a prior frozen storage (0–6-month period at −18 °C) in canned Atlantic mackerel (*S. scombrus*) that was packed under different media. As a result, no effect on the EPA value and the ω3/ω6 ratio was observed by increasing the prior storage period; however, a decrease in DHA (sunflower oil packing) and total ω3 FA (sunflower oil packing) values was detected by increasing the prior storage time, which was also observed in the current study.

### 3.4. Determination of Colour Changes

A relevant increase (*p* < 0.05) in the L* value was observed in canned fish as a result of the sterilisation step (Table 3). An additional L* value increase (*p* < 0.05) was observed in canned fish that was previously stored for 6 months under frozen conditions. This additional increase may be justified according to increased protein damage in fish corresponding to such holding conditions (i.e., extended denaturation and interaction with oxidised lipids). 

The analysis of the a* value revealed a strong decrease (*p* < 0.05) as a result of the sterilisation process (Table 3). The prior frozen storage led to higher a* values in canned horse mackerel; however, no differences (*p* > 0.05) could be observed when compared to canned fish without a prior storage period. Remarkably, an a* value decrease was reported to be correlated with haemoglobin-mediated lipid oxidation in fish and showed an inverse relationship with the secondary lipid oxidation compounds level [58,59]. Accordingly, an inverse correlation (*r* = −0.732, quadratic fitting; *p* > 0.05) between this colour index and the TBARS content was obtained in the current study.

Regarding the b* value (Table 3), a marked increase was observed after the sterilisation step. Higher average values were observed in canned samples corresponding to batches previously subjected to frozen storage; remarkably, canned fish corresponding to the 6-month storage showed significantly higher (*p* < 0.05) values than their counterpart canned samples that were not subjected to prior storage. This b* increase was explained on the basis that the formation of interaction compounds between oxidised lipids and protein-type molecules and yellowish colour development is favoured by the heating step [60,61]. According to the direct relationship between the b* parameter and the lipid oxidation development, a good correlation value was obtained in the present research between this colour index and the FR (*r* = 0.942, linear fitting; *p* < 0.05); additionally, fair correlation values were observed with the PV (*r* = 0.792, quadratic fitting; *p* > 0.05) and the TBA-i (*r* = 0.861, quadratic fitting; *p* > 0.05).

According to its great effect on the appearance and acceptability of seafood in general, the assessment of colour changes has deserved great attention. Therefore, previous research has addressed the changes produced in colour parameters as a result of the canning process. As a general behaviour, the sterilisation process has led to increased L* and b* values and to a* value decreases, and the results obtained in this study are in agreement with those previous findings. Thus, an increase in L* and b* values and a decrease in a* value in yellowfin tuna (*T. albacares*) that was packed with baby corn, green pea, and broccoli [62] and with groundnut and coconut [42] were reported. An L* value increase in sunflower oil-packed Atlantic mackerel (*S. scombrus*) [45] and an L* value increase and an a* value decrease in brine-canned Chub mackerel (*S. colias*) [41] were described. Recently, Gómez-Limia et al. [63] reported an L* and b* value increase in sunflower oil- and olive oil-canned eel (*A. anguilla*); additionally, an a* decrease was observed in sunflower oil-canned products.

Regarding the effect of prior cold storage on colour changes in canned seafood, increasing the prior storage temperature and time led to an increase in the L* value and a decrease in the a* value in canned skipjack tuna (*Katsuwonus pelamis*) [13]. Recently, Méndez et al. [23] obtained an increase in L* and b* values by increasing the prior holding time (0–6 months at −18 °C) in brine-canned horse mackerel (*T. trachurus*); however, contrary to the present research, no effect on the a* value could be inferred.

### 3.5. TMA Content

**Figure 3:**
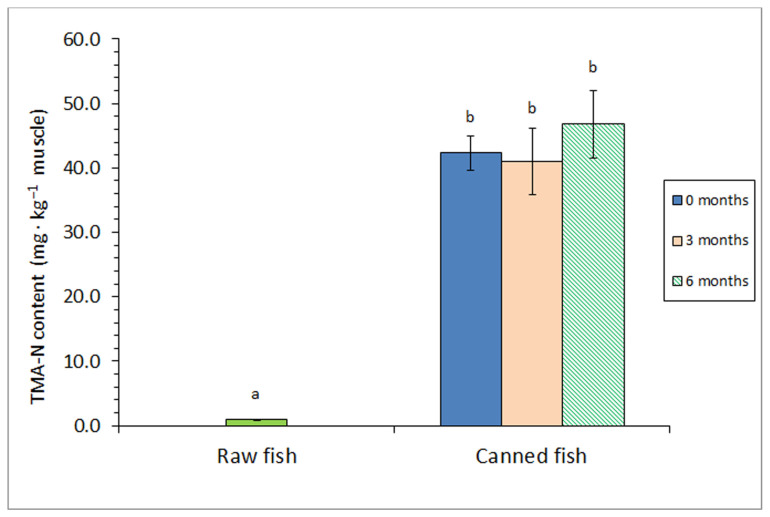
Trimethylamine (TMA) content of raw and canned horse mackerel previously subjected to different frozen storage times (0, 3, and 6 months). Average values of four (*n* = 4) replicates. Standard deviations are expressed by bars. Different letters (a,b) denote significant differences (*p* < 0.05).

A strong effect (*p* < 0.05) of the sterilisation step on the TMA content was obtained (Figure 3). Thus, all kinds of canned fish revealed higher (*p* < 0.05) levels of this deteriorative molecule than the raw fish. Notably, no differences (*p* > 0.05) in the TMA value were detected as a result of including a prior frozen storage.

TMA is considered an important deteriorative molecule whose level can indicate the degree of quality loss in seafood in general [34]. Since TMA formation as a result of microbial activity is not likely to occur during the present study, the formation of TMA should be explained by the breakdown of protein-like compounds [2,6]. Thus, previous research accounts for a relevant TMA formation as a result of the sterilisation step in canned seafood. These results have concerned different kinds of fish species and packing media such as olive oil-packed tuna (*T. thynnus*) and tomato sauce-packed sardine (*S. pilchardus*) [37]; sunflower-, groundnut-, and coconut-packed yellowfin tuna (*T. albacares*) [42]; and brine-canned Chub mackerel (*S. colias*) [41].

Concerning the effect of the prior frozen storage, previous information can be considered scarce. A marked increase in the TMA value was observed in brine-canned Atlantic Chub mackerel (*S. colias*) by increasing the prior holding period (0–15 months at −18 °C) [12].

## 4. Conclusions

The influence of different previous frozen holding periods on the quality of the canned products was analysed. A marked effect of the thermal treatment and the prior frozen storage period (3 or 6 months at −18 °C) was proved in water-packed canned horse mackerel. Thus, the sterilisation step led to a significant (*p* < 0.05) formation in the canned fish muscle of fluorescent compounds, FFAs and TMA, and to an increase in the L* and b* colour parameters; meanwhile, a decrease (*p* < 0.05) in the total ω3 FA/total ω6 FA ratio and the a* colour value were detected. The prior holding period led to an increased (*p* < 0.05) lipid oxidation (PV, TBA-i, and FR) and hydrolysis (FFA formation) development and to increased L* and b* colour values in the corresponding canned samples; furthermore, a lower (*p* < 0.05) PI and PL content were observed in canned horse mackerel previously subjected to the holding period. In most indices, quality changes were found to be higher with increased previous holding periods.

In agreement with the marked effect of the sterilisation and prior frozen storage time on the canning process of this pelagic fish species, the optimisation of such processing conditions is recommended to maximise the quality of the corresponding canned product. Regarding the thermal process, the optimisation of the time and temperature of sterilisation should be carried out, taking into account the characteristics of the raw fish composition, the packing medium, and the geometry of the canning support. On the other side, and according to the cannery’s need to hold the raw material to be employed, it is recommended to carry out the freezing step as fast as possible and maintain the storage temperature at −18 °C or even lower. 

In order to better elucidate the influence of the previous frozen holding period on the canned fish quality, further research, including physico-chemical analyses on fish samples at the frozen–thawed step, is recommended. Additionally, the effect of processing conditions on horse mackerel muscle corresponding to individuals of different characteristics (size, lipid content, catching time, etc.) than in the present study should be addressed. To guarantee that high-quality raw materials are employed for the canning process of horse mackerel, the use of traditional and complementary strategies such as glazing, the addition of natural antioxidants, and the use of impermeable packaging could constitute suitable alternatives.

## Figures and Tables

**Table 1:** Lipid oxidation of raw and canned horse mackerel previously subjected to different frozen storage times ^§^.

Lipid Quality Index	Raw Fish	Canned Fish		
		Prior Frozen Storage Time (Months)		
		0	3	6
Peroxide value (meq active oxygen·kg^−1^ lipids)	1.36 a (0.51)	1.45 a (0.32)	4.21 b (1.07)	5.91 b (1.23)
Thiobarbituric acid index (mg malondialdehyde·kg^−1^ muscle)	0.04 a (0.02)	0.12 a (0.07)	0.30 b (0.02)	0.32 b (0.03)
Fluorescence ratio	1.31 a (0.40)	3.43 b (0.54)	4.32 c (0.17)	4.89 c (0.81)

^§^ Mean values of four (*n* = 4) replicates. Standard deviations are expressed in brackets. Different letters in row (a–c) indicate significant differences (*p* < 0.05).

**Table 2:** Fatty acid (FA) parameters of raw and canned horse mackerel previously subjected to different frozen storage times ^§^.

FA Parameter ^§§^	Raw Fish	Canned Fish		
		Prior Frozen Storage Time (Months)		
		0	3	6
EPA (g·100 g^−1^ total FAs)	7.67 a (0.04)	8.26 ab (1.29)	8.79 b (0.41)	8.03 ab (0.40)
DHA (g·100 g^−1^ total FAs)	23.89 b (2.82)	19.39 ab (2.87)	17.04 a (1.71)	15.91 a (3.13)
ω3 (g·100 g^−1^ total FAs)	34.45 b (2.87)	30.50 ab (2.64)	28.74 a (1.51)	26.32 a (2.71)
ω3/ω6 Ratio	9.38 b (0.70)	7.67 a (0.79)	8.08 ab (0.71)	7.55 a (0.52)
Polyene index	1.42 c (0.12)	1.21 bc (0.09)	1.06 ab (0.07)	0.92 a (0.11)

^§^ Mean values of four (*n* = 4) replicates. Standard deviations are expressed in brackets. Different letters in row (a–c) denote significant differences (*p* < 0.05). ^§§^ Abbreviations: EPA (eicosapentaenoic acid) and DHA (docosahexaenoic acid).

**Table 3:** Colour parameters of raw and canned horse mackerel previously subjected to different frozen storage times ^§^.

Colour Parameter	Raw Fish	Canned Fish		
		Prior Frozen Storage Time (Months)		
		0	3	6
L*	42.14 a (3.19)	64.86 b (1.15)	64.03 b (0.86)	67.43 c (0.62)
a*	4.90 b (0.73)	0.77 a (0.34)	1.46 a (0.51)	0.93 a (0.25)
b*	3.13 a (0.07)	12.81 b (1.63)	14.25 bc (2.33)	16.47 c (1.32)

^§^ Mean values of four (*n* = 4) replicates. Standard deviations are expressed in brackets. Different letters in row (a–c) denote significant differences (*p* < 0.05).

## Data Availability

Data are contained within the article.
